# Decreased Expression of C10orf10 and Its Prognostic Significance in Human Breast Cancer

**DOI:** 10.1371/journal.pone.0099730

**Published:** 2014-06-17

**Authors:** Junjiang Deng, Yan Dong, Chong Li, Wenwei Zuo, Gang Meng, Chengping Xu, Jianjun Li

**Affiliations:** 1 Department of Oncology, Southwest Hospital, Third Military Medical University, Chongqing, China; 2 Cadre’s Sanatorium, Third Military Medical University, Chongqing, China; 3 Department of Pathology, Southwest Hospital, Third Military Medical University, Chongqing, China; The University of Hong Kong, China

## Abstract

Breast cancer is a common malignant tumor, which severely threatens the health of women with an increasing incidence in many countries. Here, we identified C10orf10 as a novel differentially expression gene using expression microarray screening. The expression analysis indicated that C10orf10 was frequently decreased in human breast cancers compared to noncancerous breast tissues (81/95, P = 0.0063). Kaplan-Meier analysis indicated that patients with low C10orf10 expression showed a poorer prognosis both in mRNA (n = 1115, P = 0.0013) and protein (n = 100, P = 0.003) levels. Univariate and multivariate analysis showed that the C10orf10 expression was an independent prognostic factor for overall survival of breast cancer patients. Further analysis revealed that low expression of C10orf10 was an unfavorable factor for the prognosis of the patients who were luminal A, luminal B, Her2+ subtypes, at histological grade 2, lymph node negative and ER positive. Our data provided the first evidence that C10orf10 expression was frequently decreased in breast cancer tissues, and low expression of C10orf10 may be an important prognostic factor for poorer survival time of breast cancer patients.

## Introduction

Breast cancer (BC), the top cancer in women both in the developed and developing countries, is a leading cause of cancer-related death worldwide and is particularly spreading in China at an alarming rate [1], [2]. Moreover, increasing young women are suffering from this disease, and most of them are diagnosed with advanced stage. Although early detection and effective treatment have decreased BC mortality in recent years, prevention and therapy of BC remain a major public health concern [3]. Breast cancer mortality can be reduced by early diagnosis and finding prognostic factors. The latter strategy can be employed to predict the outcome of the individual patient and select appropriate therapy [4]. Therefore, identifying reliable prognostic factors is urgent in BC [5]–[7].

At present, prognosis is mainly based on clinicopathological parameters such as lymph node status, tumor size, distant metastasis, histological type and grade [8]–[10]. These are powerful prognosticators, but these may be only rough measures of the biological behavior of a tumor. Moreover, some of these parameters might be influenced by the subjectivity of the pathologist and limited in their prognostic value [11], [12]. Thus, it is valuable to find other prognostic markers, which can be measured reliably to support these traditional factors, and can then be used to help in evaluating patient’s risks and selection of treatment [13], [14].

Chromosome 10 open reading frame 10 (C10orf10, also called DEPP) was initially cloned from the human endometrial stromal cells cDNA library enriched for progesterone-induced genes [15]. C10orf10 is highly expressed in various tissues including ovary, kidney, placenta and liver [15]–[17]. It is also detected in endothelial cells of peripheral tissues [17], and is regulated by FoxO1 and FoxO by binding to IRS element in its promoter [18], [19]. Increased expression of C10orf10 is observed in several conditions. Progesterone or androgen induces C10orf10 mRNA in endometrial stromal cells [15], and it is also induced in a malignant glioma cell line in response to hypoxic stress [20]. Recently, C10orf10 is reported to be upregulated by feeding in insulin-sensitive tissues including white adipose tissue and liver [16], and is upregulated in subsets of endothelial cells in settings of adult neo-vascularization including tumor angiogenesis [17]. However, the expression and clinical significance of C10orf10 in cancer remains largely unclear.

In the present study, we identified C10orf10 as a novel differentially expression gene in normal and tumor breast tissues using gene expression microarray screening. The screening result was confirmed by RT-PCR, qRT-PCR and immunohistochemical staining (tissue microarrays) in normal breast, BCs and corresponding noncancerous breast tissues. Then, the prognostic significance of C10orf10 was analyzed in human BCs. The data showed that C10orf10 expression is frequently lower in BC tissues, and this low expression may be an important prognostic factor for BC patients.

## Materials and Methods

### Patient Samples

A total of 120 BC patients and 85 controls (including 75 matched tumor and corresponding noncancerous breast tissues) were recruited from the Southwest hospital in Chongqing, China. This study was approved by the ethics committee of the Southwest hospital in Chongqing, China. Informed consent was signed by all of the recruited patients.

### Isolation of Total RNA

Total RNAs were extracted from frozen tissues by homogenization with a power homogenizer in TRIzol Reagent according to the manufacturer’s protocol. Thereafter, total RNAs (2.0 µg) were treated with DNase I to eliminate the genomic DNA contamination, and then were reverse-transcribed to generate cDNAs.

### Analysis of C10orf10 Expression by RT-PCR and qRT-PCR

The expression level of C10orf10 was determined by the reverse transcription polymerase chain reaction (RT-PCR), and human β-actin was amplified as an endogenous control. A series of PCRs with different cycles were performed to determine the linear phase for RT-PCR. Based on these pilot experiments, the appropriate cycles were chosen. The primers for C10orf10 and β-actin are as follows: C10-F, 5′-GCCTGGATGACTACGTGAGG-3′; C10-R, 5′-ACTGCCCAAAAGTCC.

AGCTT-3′; Actin-F, 5′-TTCTACAATGAGCTGCGTGTG-3′; Actin-R, 5′-GGGGTG.

TTGAAGGTCTCAAA-3′. The Real-time Quantitative RT-PCR (qRT-PCR) was performed using an iQ5 real-time detection system (Bio-Rad Laboratories, Hercules, CA, USA) and SYBR Premix Ex TaqTMII (Takara). The relative gene expression was calculated by the equation 2^−ΔΔCT^. The sequences of the primers are as follows: C10-F2, 5′-AAGCTGGACTTTTGGGCAGT-3′; C10-R2, 5′-GTTCATGGATCAC-.

CGGGAGG-3′; Actin-F2, 5′-TGACGTGGACATCCGCAAAG-3′; Actin-R2, 5′-CTGGAAGGTGGACAGCGAGG-3′. All qRT-PCRs were performed in triplicate.

### Tissue Microarray Generation

All samples from BC patients were reviewed histologically by hematoxylin and eosin staining, and two cores were taken from each representative tumor tissue and from adjacent tissue within a distance of 10 mm (the size of the tissue was not allowed for a non-cancerous adjacent tissue taking a few centimeters away from the tumor. The non-cancerous adjacent tissues were obtained compared with normal tissue, and were reviewed histologically by hematoxylin-eosin staining and pathological referral by at least two pathologists [21]) to construct the tissue microarray (TMA) slides (in collaboration with Shanghai Biochip Company Ltd, Shanghai, China). Duplicate cylinders from two different areas, intratumoral and peritumoral (a total of four punches), were obtained. Then, a TMA section with pairs of tumors and matched peritumoral tissues were constructed [21].

### Immunohistochemical (IHC) Analysis

Immunohistochemical staining was performed using the antibody against C10orf10 (1∶100; Santa Cruz Biotechnology) as described previously [22]. The staining was evaluated for the tumor cells. The immunostaining was considered positive when ≥10% of the tumor cells being immunoreactive. The percentage of positive cells, as the extent of immunostaining, was quantified classified into 5 groups under microscope: <10% positive cells for 0; 10% to 25% for 1; 26% to 50% for 2; 51% to 75% for 3 and ≥76% for 4. The intensity of staining was graded as negative (scored as 0), weak (1), moderate (2) or strong (3) positivity. The two pathologists independently reviewed all core biopsies. The sum of the percentage of positive staining and the intensity was used to define expression levels. The dataset has been submitted to the Gene Expression Omnibus (GEO), and the accession number is GSE56985.

### Statistical Analysis

Statistical analyses were performed by the SPSS 16.0 software (SPSS, Inc., Chicago, IL). The results were expressed as the mean ± standard error (SE). Measurement data were analyzed by Student’s t test. The chisquare test was used to analyze the differences of categorical variables. Clinical and pathologic characteristics of the patients were compared by Pearson Χ2 test. Overall survival (OS) was calculated according to the Kaplan-Meier method and evaluated by the log-rank test. A Kaplan-Meier survival database that contains survival information of BC patients and gene expression data obtained by using Affymetrix microarrays was used [23]. The probe set is 209183_s_at (There are two C10orf10 probe sets, 209182_s_at at the open reading frame and 209183_s_at at the 3′ un-translated region. Due to a different alternatively spliced isoform of C10orf10, FIG No. AB025244, sharing 209182_s_at, we chose the probe set of 209183_s_at in the present study) and split patients by median or auto select best cutoff. Cox regression was performed for multivariate analysis of prognostic predictors. P<0.05 was considered significant.

## Results

### C10orf10 is Frequently Decreased in Human BC Tissues

**Figure 1:**
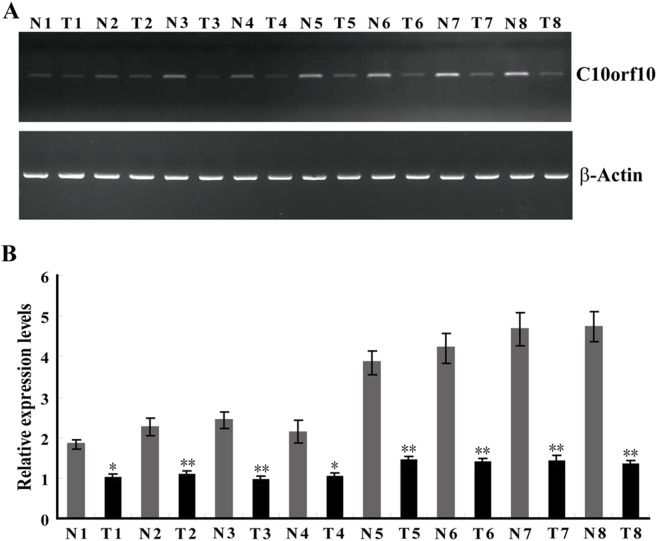
The mRNA expression of C10orf10 is frequently decreased in human BC tissues. (A) RT-PCR analysis of C10orf10 mRNA levels in normal breast and BC tissues. The β-actin was used as an internal control. (B) C10orf10 mRNA levels was also detected by qRT-PCR analysis in normal breast and BC tissues. The β-actin was used as an internal control. Error bars indicate s.d. (n = 3).

**Figure 2:**
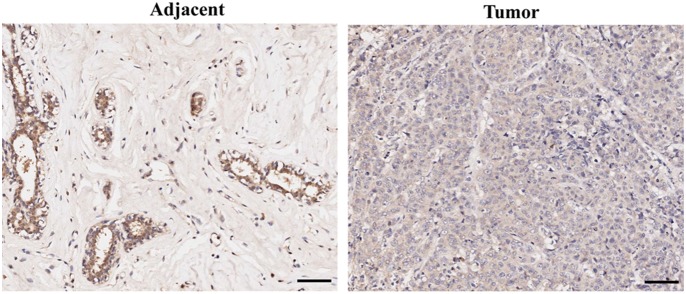
The representative protein expression of C10orf10 in pairs of BC and corresponding noncancerous breast tissues. Immunohistochemistry analysis of C10orf10 expression in 75 tumor and corresponding noncancerous samples, and C10orf10 is expressed in epithelial cells of corresponding noncancerous breast tissues (mainly expressed in the cytoplasm, and also mildly expressed in some part of the nucleus). C10orf10 expression is decreased in most BC tissues compared to corresponding noncancerous breast tissues. Immunohistological staining was performed with an anti-C10orf10 antibody, and scale bars are 100 µm.

To investigate the level of C10orf10 expression in human BCs, RT-PCR and qRT-PCR assays were performed in 10 normal breast (10 different normal healthy breasts) and 20 BC tissues (20 different breast cancers). The results showed that the expression of C10orf10 in most cases (17/20) of BC tissues was downregulated compared to the mean expression in 10 normal breast tissues (Figure 1A, 1B), and the mean expression level of C10orf10 in BC tissues (1.0±0.13) was remarkably lower than that in normal breast tissues (2.73±0.28; P = 0.0063) (Figure 1B). To determine whether the protein level of C10orf10 is altered, we evaluated C10orf10 protein expression by immunohistochemistry on a tissue microarray (TMA) slide containing 75 pairs of BC and corresponding noncancerous breast tissues. The data of TMA indicated that C10orf10 protein was decreased in most (64/75) BC tissues compared to noncancerous breast tissues (Figure 2).

### Low Expression of C10orf10 is Significantly Associated with Poor OS in BC Patients

**Figure 3:**
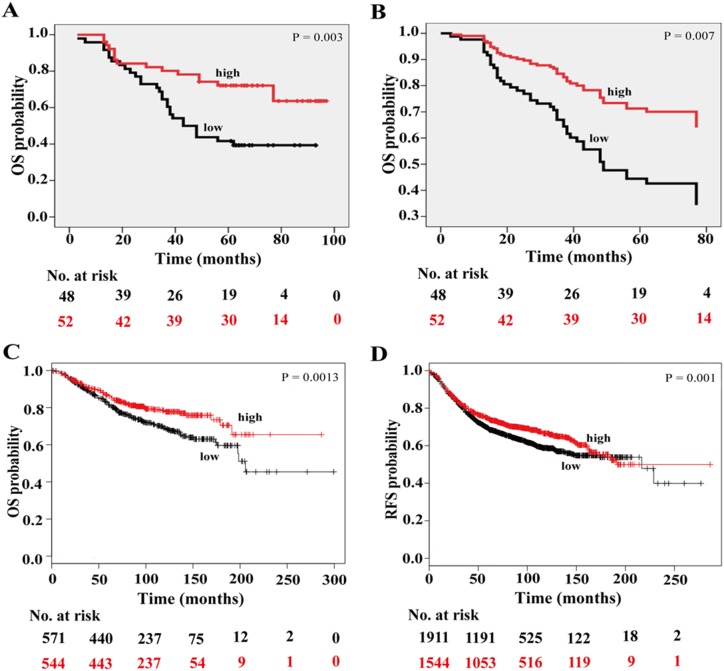
Low C10orf10 expression is correlated with shorter survival in BC patients. (A) Survival analysis of C10orf10 protein expression in 100 BC patients by Kaplan-Meier survival curve. Tissue array analysis was performed in 100 cases of patients with the survival information. The patients with low expression had a poorer overall survival than those with high expression; HR, hazard ratio; low, staining negative and weak; high, staining moderate and strong. (B) Survival analysis of C10orf10 expression in 100 BC patients by multivariate Cox regression. The C10orf10 expression is an independent prognostic factor. (C) Kaplan-Meir survival analysis of C10orf10 mRNA in 1115 BC patients with Kaplan-Meier Plotter (http://kmplot.com/analysis/index). The overall survival was longer in the *C10orf10* high expression group than in the *C10orf10* low expression group. Auto select best cutoff was chosen in the analysis; Cutoff value used was 1086; Expression range of the probe was 143–3881. (D) Kaplan-Meir survival analysis of C10orf10 mRNA in 3455 BC patients with Kaplan-Meier Plotter. The relapse-free survival was longer in the *C10orf10* high expression group than in the *C10orf10* low expression group. Auto select best cutoff was chosen in the analysis; Cutoff value used was 1088; Expression range of the probe was 32–5515.

Decreased expression of C10orf10 in BCs suggests that it may be associated with BC development. To investigate the correlation between C10orf10 expression and survival of BC patients, we scored the specific staining of the immunohistochemical TMA containing 100 BC patients by intensity and percentage of positive staining, combining negative with low, and mediate with high expression to obtain two groups, low and high, respectively. Survival analysis by Kaplan-Meier and log rank test demonstrated that the patients with high expression of C10orf10 protein had longer OS time than these with low expression (P = 0.003) (Figure 3A). To avoid the influence caused by univariate analysis, the C10orf10 expression as well as other parameters was examined in multivariate Cox regression analysis (adjusted for age, stage, grade, tumor size and lymph node). The C10orf10 protein expression was found to may be an independent prognostic factor (hazard ratio (HR)  = 0.418, P = 0.007) in addition to age (HR = 1.044, P = 0.012) and lymph node (HR = 1.089, P = 0.017) (Figure 3B, Table 1). When investigating the association between C10orf10 expression and clinicopathologic features of BC patients, there were no significant correlations between C10orf10 expression and age, stage, grade and tumor size (Table 2). However, there was a significant correlation between C10orf10 expression and lymph node status (P = 0.045) (Table 2).

**Table 1:** Multivariate analysis of different prognostic factors in BC patients.

Expression level	Variable	HR	95% CI	P value
The protein level expression of C10orf10	Age	1.044	1.009–1.079	0.012
	Stage	1.287	0.815–2.032	0.279
	Grade	0.915	0.522–1.602	0.755
	Tumor size	0.982	0.785–1.228	0.871
	Lymph node	1.089	1.015–1.169	0.017
	C10orf10 expression	0.418	0.221–0.791	0.007

Abbreviations: HR, hazard ratio; CI, confidence interval.

**Table 2:** Correlations of C10orf10 expression with clinicopathologic features of BC patients.

		C10orf10 Expression		
Clinical Feature	Total	High (n = 52)	Low (n = 48)	P value
Age (years)				
<60	52	27	25	1.00
≥60	47	25	22	
Stage				
I+II	58	31	27	0.84
III	42	21	21	
Grade				
1	14	7	7	0.795
2	56	31	25	
3	29	14	15	
Tumor size				
≤3 cm	47	28	19	0.167
>3 cm	53	24	29	
Lymph node				
Negative	51	32	19	0.045
Positive	48	20	28	

### C10orf10 is a Potential Prognostic Factor for BC Patients

As indicated above, TMA studies showed a significantly correlation between C10orf10 expression and BC prognosis. To further validate the association between C10orf10 expression and prognostic outcomes of the BC patients, we then examined the contribution of C10orf10 mRNA expression, which is correlated with C10orf10 protein expression (data not shown), to the OS of BC patients in a clinical microarray database [23]. This database collected gene expression data that were obtained by using Affymetrix microarrays and the OS information of 1115 BC patients. The OS analysis revealed that high expression of C10orf10 mRNA indeed predicts better survival of the BC patients (HR = 0.67, P = 0.0013) (Figure 3C). These results indicated that the C10orf10 expression could act as a potential marker for prognosis evaluation of BCs. In addition, BC patients with high C10orf10 expression also had a better relapse-free survival (RFS) compared with the low group (HR = 0.82, P = 0.001) (Figure 3D).

### Low Expression of C10orf10 Predicts Poorer Survival of the Patients with Luminal A, Luminal B and Her2+

**Figure 4:**
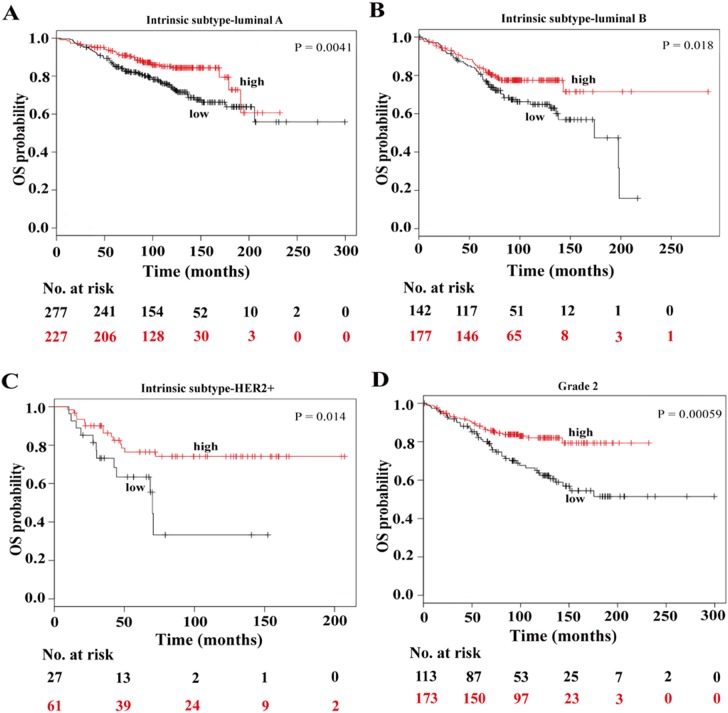
Low expression of C10orf10 is correlated with shorter survival in patients from KM plotter with luminal A, luminal B, Her2+ and grade 2 of the breast cancer. (A) Kaplan-Meir survival analysis of C10orf10 in 504 patients with luminal A tumors. Auto select best cutoff was chosen in the analysis; Cutoff value used was 1117; Expression range of the probe was 186–3881. (B) Kaplan-Meir survival analysis of C10orf10 in 319 patients with luminal B tumors. Auto select best cutoff was chosen in the analysis; Cutoff value used was 1052; Expression range of the probe was 181–3158. (C) Kaplan-Meir survival analysis of C10orf10 in 88 patients with Her2+ tumors. Auto select best cutoff was chosen in the analysis; Cutoff value used was 655; Expression range of the probe was 153–3475. (D) Survival analysis of C10orf10 expression in 286 patients with grade 2 of the breast cancer. Auto select best cutoff was chosen in the analysis; Cutoff value used was 1117; Expression range of the probe was 143–3859.

Based on well-characterized molecular features, BC could be categorized into four subtypes, basal-like, luminal A, luminal B, and Her2+ tumors, which exhibit distinct oncogenic activation pathways, tumor progression pattern and prognostic outcomes [24], [25]. To determine the correlation between C10orf10 expression and the survival times of BC patients with different subtypes, we then analyzed survival data of each tumor subtypes by stratifying the patients based on the C10orf10 expression levels. The results indicated that C10orf10 expression was not statistically associated with the OS times of patients who have basal-like BC (HR = 0.65, P = 0.13). However, there was a statistically significant effect of low C10orf10 expression for the poorer survival of the patients who have luminal A, luminal B and Her2+ BC (HR = 0.56, P = 0.0041; HR = 0.61, P = 0.018 and HR = 0.39, P = 0.014, respectively) (Figure 4A–4C). These data suggest that low expression of C10orf10 may be an unfavorable factor for the prognosis of luminal A, luminal B and Her2+ tumors.

### Low Expression of C10orf10 Predicts Poorer Survival of the Patients with Grade 2 of the BC

To determine the association between C10orf10 expression and the OS times of BC patients with different grades, we also analyzed survival data of each grades by stratifying the patients based on the C10orf10 expression levels. The results indicated that C10orf10 expression was not statistically associated with the OS times of BC patients at grade 1 (HR = 0.79, P = 0.61) and grade 3 (HR = 0.76, P = 0.18). While, there was a statistically effect of low C10orf10 expression on the poorer OS of the BC patients at grade 2 (HR = 0.45, P = 0.00059) (Figure 4D). These findings suggest that low expression of C10orf10 is an unfavorable factor for the prognosis of the BC at grade 2.

### Low Expression of C10orf10 Predicts Poorer Outcomes of the BC Patients with Lymph Node Negative and ER Positive

**Figure 5:**
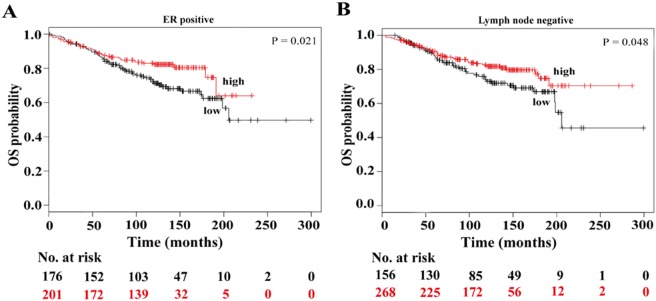
Low expression of C10orf10 is correlated with shorter survival in BC patients from KM plotter with ER positive and lymph node negative. (A) Survival analysis of C10orf10 expression in 377 BC patients with ER positive. Auto select best cutoff was chosen in the analysis; Cutoff value used was 1117; Expression range of the probe was 143–3356. (B) Survival analysis of C10orf10 expression in 424 BC patients with lymph node negative. Auto select best cutoff was chosen in the analysis; Cutoff value used was 923; Expression range of the probe was 143–3356.

Now there is still a compelling question that whether low expression of C10orf10 also predict poorer survival of the BC patients with different lymph node statuses and ER statuses. We observed a statistically effect of low C10orf10 expression on the poorer survival of the BC patients who were lymph node negative (HR = 0.66, P = 0.048) and ER positive (HR = 0.61, P = 0.021) (Figure 5A, 5B), but no effect on that of the patients who were lymph node positive (HR = 0.63, P = 0.089) and ER negative (HR = 0.62, P = 0.095).

## Discussion

C10orf10 has been reported to be expressed in various tissues of human, and its expression is closely related to hypoxic stress and progesterone or androgen [15]–[17], [20]. However, the expression of C10orf10 in cancers has not been reported up to date. In the present study, we first detected the expression of C10orf10 in 10 normal breast tissues, 20 BC tissues (mRNA level), 75 pairs of BC and corresponding noncancerous breast tissues, and other 25 BC tissues (protein level). The data showed that the relative expression level of C10orf10 in BC was frequently lower than that in noncancerous breast tissues and normal breast tissues, suggesting that it might be associated with BC development. Further studies are required to analyze the functional roles of C10orf10 in human BCs.

As different cancer treatments are effective in different subgroups of patients, there is a tremendous need for novel predictive and prognostic markers to improve the outcome of cancer patients [26]. BC is the most common malignant disease in women worldwide, and is a group of heterogeneous diseases showing various biological and clinical characteristics. Patient management is currently based on easily identifiable clinical and pathological characteristics, but these only partially reflect disease heterogeneity. Many principal factors, such as age, status of lymph node and HER2, tumor size, have been used in predicting the outcome of BC patients [27], [28]. However, their roles in determining the individual risk of the patient are limited. Therefore, it is still needed to exploit clinically useful, readily available prognostic markers in the management of BC. In the present study, we found that the expression of C10orf10 was decreased in most BCs, which suggested that it might be associated with BC development. Then, we analyzed the clinicopathologic and prognostic significance of C10orf10 expression in 100 BC patients at protein level and 1115 BC patients at mRNA level. Our data showed that the patients with low C10orf10 expression both in protein and mRNA levels had significantly poor OS using the Kaplan-Meier method and log-rank test. Multivariate analysis indicated that C10orf10 protein expression might be an independent prognostic factor for OS in BC patients. These results suggest that C10orf10 expression is significantly associated with a poor prognosis independently of other factors. It is the first time to report that C10orf10 expression can be used as a novel diagnostic marker for poor prognosis in cases of BC. In addition, previous study has revealed that the value of events per variable (EPV)  = 10 seems most prudent [29], however, the EPV value is below this in our study. Thus, the results of multivariate Cox regression analyses may not be so accurate, and it needs to be validated in a larger series.

To further determine the association between C10orf10 expression and survival time of BC patients with different clinical subtypes, histological grades, lymph node statuses and ER statuses, survival data of each situation was analyzed by stratifying the patients based on the C10orf10 expression levels. The results showed that low expression of C10orf10 might be an unfavorable factor for the prognosis of BC patients who were luminal A, luminal B, Her2+ subtypes, at histological grade 2, lymph node negative and ER positive. Further, the effect of C10orf10 mRNA levels on OS observed in patients with luminal and Her2+ tumors might be due to the much lower C10orf10 mRNA levels in these subtypes than in the basal-like tumors.

Although the log rank p-values are significant, the lines intersect in some survival plots, such as Figures 3D, 4A, and 5A. Considering that it is OS, the patients may die due to other causes. The follow-up times for some survival curves are also different, which may be due to some missing cases because of incomplete information in multivariate Cox regression analyses. Besides, in the present study, there is an effect on OS in patients with luminal A and luminal B tumors, and it is not surprising that an effect on OS is observed in both ER positive and grade 2 patients because the majority of these are comprised of patients with luminal A and B tumors.

Taken together, the present study indicates that C10orf10 is downregulated in BC tissues and may be a novel independent molecular marker for predicting the outcome of BC patients. However, further investigation of the cell biology of C10orf10 and its potential as a therapeutic target in BC is clearly warranted.
